# Quand le dentier devient danger!

**DOI:** 10.11604/pamj.2017.27.179.12902

**Published:** 2017-07-05

**Authors:** Nouzha Sadak, Laila Herrak, Leila Achachi, Mustapha El Ftouh

**Affiliations:** 1 Service de Pneumologie, CHU Ibn, Sina, Rabat, Maroc; 2 Faculté de Médecine et de Pharmacie, Université Mohammed V, Rabat, Maroc

**Keywords:** Corps étrangers, bronchoscopie, extraction, adulte, Foreign body, bronchoscopy, extraction, adult

## Abstract

Bien que rare chez l'adulte, l'inhalation de corps étranger (CE) est un accident grave pouvant mettre en jeu le pronostic vital ou entrainer des séquelles importantes. Nous rapportons l'observation d'un patient de 50 ans, sans antécédents pathologiques particuliers, qui s'est présentée aux urgences pour douleur thoracique, toux intermittente et dyspnée d'effort, six jours après avoir inhalé, accidentellement, sa prothèse dentaire en plastique lors d'un repas. L'examen clinique était sans particularités. La radiographie thoracique ainsi que l'ASP ne montraient pas d'anomalie. Une bronchoscopie souple sous anesthésie générale a permis de visualiser le CE au niveau du tronc intermédiaire et l'extraction a été réalisée avec succès évitant ainsi le recours à un geste beaucoup plus invasif. La radiographie standard peut s'avérer utile en visualisant les CE radio-opaque ou par des signes indirects évoquant la présence d'un CE, mais le recours à la bronchoscopie à visée diagnostique et thérapeutique est primordial.

## Abstract (en)

Although rare in adults, foreign body aspiration (FBA) is a serious accident which can be potentially life threatening or lead to significant sequelae. We report the case of a 50 year old patient without previous pathological history, presenting to the emergency department with chest pain, intermittent cough and exertional dyspnea occurring six days after the accidental aspiration of his plastic dental prosthesis during a meal. Clinical examination was unremarkable. Chest X-ray as well as abdominal x-ray requiring no prior preparation showed no abnormalities. Flexible bronchoscopy under general anesthesia showed FBA at the level of the intermediate trunk. Successful extraction was performed avoiding a much more invasive procedure. Standard X-ray can be useful to visualize radio-opaque FBA or indirect signs suggesting the presence of FBA, but diagnostic and therapeutic bronchoscopyis is essential.

## Introduction

L'inhalation de corps étranger (CE) est un accident rare chez l'adulte mais néanmoins grave, pouvant mettre en jeu le pronostic vital ou entrainer des complications parfois lourdes de conséquences.

## Patient et observation

**Figure 1:**
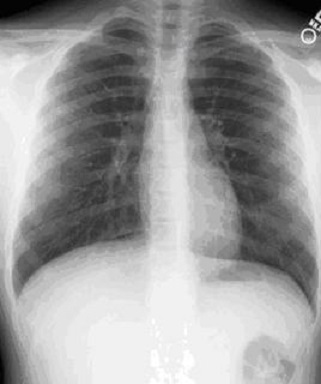
Radiographie thoracique normale

**Figure 2:**
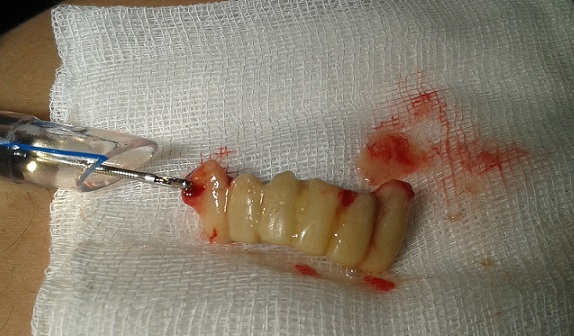
Dentier en résine

Il s'agit d'un patient de 50 ans, sans antécédents pathologiques particuliers. Six jours après avoir inhalé accidentellement sa prothèse dentaire en résine au cours d'un repas, le patient s'est présentée aux urgences dans un tableau de dyspnée d'effort d'installation brutale avec toux intermittente et douleur thoracique; dans un contexte d'apyrexie et de conservation de l'état général. L'examen clinique était sans particularités. La radiographie thoracique (Figure 1) ne montrait pas d'anomalie. Une bronchoscopie souple sous anesthésie générale a permis de visualiser le CE au niveau du tronc intermédiaire et l'extraction a été réalisée avec succès évitant ainsi le recours à un geste beaucoup plus invasif (Figure 2).

## Discussion

L'inhalation accidentelle de corps étranger est l'apanage de l'enfant avec une prédominance masculine [1]. Chez l'adulte, les corps étrangers intra-bronchiques sont rares, de diagnostic souvent difficile et constituent une urgence diagnostique et thérapeutique [2]. Les CE les plus communément rencontrés sont organiques (aliments), billes, pièces de monnaie, prothèses dentaires et objets métallique [3]. Cependant, les dernières années ont connu l'émergence d'un nouveau type de corps étranger, spécifique au monde islamique: l'épingle a foulard, en raison de la tradition socio- culturelle et religieuse [4–7]; en particulier chez les jeunes adolescentes [8]. Chez l'enfant, le risque d'inhalation de corps augmente avec l'acquisition de la pronation et la tendance à porter les objets à la bouche. Lors des repas cet accident est favorisé par une mastication moins efficace et le manque de coordination entre la déglutition et la fermeture de la glotte [3, 9, 10]. À l'âge adulte, la diminution de la vigilance, les émotions brutales au cours des repas et l'exercice de certains métiers (couturiers, cordonniers, menuisiers) sont des causes fréquentes d'inhalation des corps étrangers intra-bronchiques; on note une prédisposition particulière du sujet âgé aux fausses routes pendant le sommeil à cause de la mauvaise denture, de la diminution du réflexe tussigène et de la déglutition [1, 3]. L'élément clé du diagnostic est la survenue d'un syndrome de pénétration fait d'accès de suffocation avec quintes de toux avec parfois cyanose, tirage ou cornage, pouvant engager le pronostic vital. Cependant, ce syndrome peut manquer ou être méconnu pendant des années, responsable d'un retard diagnostique [2, 3]. En effet, la négligence d'un corps étranger intra-bronchique favorise son enclavement, entrainant des manifestations respiratoires trainantes et récidivantes. La chronicité des phénomènes inflammatoires aboutit à la formation d'un bourgeon qui recouvre le corps étranger, gênant sa visualisation à l'endoscopie [1, 2].

La radiographie du thorax est d'un grand apport diagnostique, cependant la visualisation du corps étranger dépend de sa localisation, sa nature, sa consistance, son aspect et sa taille (2); certains signes radiologiques indirects comme l'atélectasie ou l'emphysème obstructif permettent d'évoquer le diagnostic [3]. Dans notre cas, la nature radio-transparente et l'absence de signe radiologiques évocateurs n'ont pas permis l'identification du CE. Même devant un bilan clinico-radiologique normal, une fibroscopie bronchique à visée diagnostique est indispensable en cas de suspicion de corps étranger bronchique, afin d'éviter l'installation des lésions parenchymateuses [2]. La stratégie thérapeutique pour l'extraction d'un CE trachée-bronchique n'est pas encore consensuelle et dépend étroitement de l'expérience des équipes. La bronchoscopie rigide sous anesthésie générale demeure la procédure de choix [3, 11]; toutefois, elle comporte des inconvénients tels que la nécessité d'une anesthésie générale et le risque de morbidité supplémentaire chez les patients âgés [12]. La fibroscopie souple connaît un engouement certain car elle ne nécessite pas une anesthésie générale et permet une meilleure accessibilité des voies aériennes distales et lobes supérieurs; de plus l'utilisation de diverses pinces à préhension, pince paniers, cathéters à ballonnet, extracteurs magnétiques a permis d'améliorer la technique; toutefois, le succès de l'extraction des corps étrangers utilisant la bronchoscope flexible dépendra en grande partie de l'expérience et la compétence de l'opérateur [12, 13]. En cas d'échec de l'extraction endoscopique, la chirurgie, radicale ou conservatrice peut être indiquée en dernier recours [2, 3]. Dans notre cas l'extraction par bronchoscopie souple sous AG a été réalisée avec succès, évitant au patient le recours à la chirurgie.

## Conclusion

L'inhalation des corps étrangers intra-bronchiques peut conduire à des destructions parenchymateuses irréversibles, la meilleure stratégie thérapeutique reste la prévention.

## Conflits d’intérêts

Les auteurs ne déclarent aucun conflit d'intérêt.
